# Low expression of hsa_circ_0001811 in gastric cancer and its role in clinical diagnosis

**DOI:** 10.1002/jcla.23642

**Published:** 2020-11-07

**Authors:** Haiyan Zhang, Zhe Li, Yao Ruan, Weiliang Sun, Rui Yu

**Affiliations:** ^1^ Department of Biochemistry and Molecular Biology Zhejiang Key Laboratory of Pathophysiology Medical School of Ningbo University Ningbo China; ^2^ Fuyang Fifth People’s Hospital Fuyang China; ^3^ The Affiliated People’s Hospital Ningbo University Ningbo China

**Keywords:** biomarker, circRNAs, gastric cancer, hsa_circ_0001811

## Abstract

**Background:**

As far as gastric cancer is concerned, there is a lack of specific early diagnostic biomarkers in clinical diagnosis. Circular RNAs (circRNAs) have been found to be stable in gastric cancer tissues and plasma, so they have the potential to become new type of diagnostic biomarkers for gastric cancer.

**Materials and Methods:**

The hsa_circ_0001811 expressions in gastric cancer tissues and paired non‐cancer tissues, preoperative and postoperative plasma of patients with gastric cancer, and plasma of healthy volunteers were detected using quantitative reverse transcription‐polymerase chain reaction. The receiver operating characteristic (ROC) curves were drawn; and the combined ROC curves were used to analyze their diagnostic values. The correlation between the plasma or tissue hsa_circ_0001811 levels and the clinicopathological factors of gastric cancer was further analyzed.

**Results:**

Hsa_circ_0001811 was found to be lowly expressed in gastric cancer tissues and plasma from patients with gastric cancer. The area under the ROC curve (AUC) in the gastric cancer tissues and the plasma of patients with gastric cancer was 0.658 and 0.747, respectively. The AUC increased to 0.824 after combination of both. The expression level of hsa_circ_0001811 in gastric cancer tissue correlated with carcinoembryonic antigen (CEA) (*P* = .0347), tissue differentiation (*P* = .0138), and lymph node metastasis (*P* = .0234), while plasma hsa_circ_0001811 level was related to carbohydrate antigen (CA19‐9) (*P* = .0278), lymph node metastasis (*P* = .0469), distant metastasis (*P* = .0384), and age (*P* = .0085).

**Conclusions:**

The above results indicate that hsa_circ_0001811 may become a new biomarker for clinical diagnosis of gastric cancer.

## Graphical abstract

Sanger sequence results of qRT‐PCR products of hsa_circ_0001811 are consistent with the sequence of target circRNA. Compared with paired non‐cancer tissues, hsa_circ_0001811 was low expressed in gastric cancer tissues (2A); and the expression of plasma specimens one day before surgery in gastric cancer patients was lower, then those in ten days after surgery and plasma samples of healthy volunteers (2B). The AUC of hsa_circ_0001811 in gastric cancer tissues was 0.658, while the AUC in plasma was 0.747. When tissues and plasma were combined used, the AUC increased to 0.824, further showing the higher diagnostic value of hsa_circ_0001811.

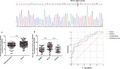

## 1 INTRODUCTION

Gastric cancer is one of the global mortality diseases and has a poor prognosis due to the lack of ideal tumor biomarkers.^1^ The deaths caused by gastric cancer account for about a quarter of all the deaths from malignant tumors.^2^ It is a disease that seriously threatens people's health. The early symptoms of gastric cancer are usually not obvious, such as abdominal pain, belching, and regurgitation.^3^ Generally, the symptoms will be alleviated or relieved after taking drugs, so it is often ignored and not diagnosed.^4^ Therefore, it is particularly important to search for new markers for early diagnosis of gastric cancer.^5^

Circular RNAs (circRNAs) are a type of new endogenous RNA produced by non‐canonical reverse splicing events.^6^ They have no free 5 'and 3' ends and no poly‐A tail structure, but form a circular structure with covalent bonds.^7^ Therefore, compared with linear RNAs, circRNAs are not easily degraded by exonuclease, can exist stably in vivo,^8^ and are highly conservative, indicating that circRNAs have obvious advantages as a new diagnostic biomarker.^9^ In addition to stability and conservation, the abundance and tissue‐specific expression of circRNA also make it an obvious advantage as a new diagnostic biomarker.

In our previous study, we used circRNA microarray to detect the expression profile of circRNAs in gastric cancer tissues and matched non‐cancer tissue samples.^10^ hsa_circ_0001811 is one of the significant differently expressed circRNAs between gastric cancer tissues and matched non‐cancer tissues. The gene of hsa_circ_0001811 is located at 8q21.11 (chr8:74585342‐74601048), and its related gene symbol is staufen double‐stranded RNA binding protein 2 (*STAU2*).

## 2 MATERIALS AND METHODS

### 2.1 Clinical specimens and pathological data

In this study, 100 pairs of gastric cancer tissues and matched non‐cancer tissues, 42 preoperative and postoperative plasma samples of gastric cancer patients, and 42 healthy volunteer plasma samples were obtained from Affiliated People's Hospital of Ningbo University. All tissue specimens were obtained by experienced surgeons. Tissues were diagnosed by pathology, and the tumor staging is based on the International Cancer Alliance's Tumor‐Node‐Metastasis (TNM) staging system. The histological grade was evaluated according to the National Comprehensive Cancer Network (NCCN) oncology clinical practice guidelines (V.1.2011).^11^ Immediately after obtaining, the tissues were put into RNA‐fixer Reagent (Kang Wei) and then stored at −80℃ until use. The patients involved signed an informed consent, and the study was approved by the Ningbo University Medical Ethics Committee.

### 2.2 Total RNA extraction

When extracting RNA from all tissue samples, the soybean size was taken, crushed and abstracted by TRIzol reagent (Invitrogen), while plasma samples were abstracted by TRIzol LS reagent (Invitrogen). Then, the concentration and purity of RNA were measured via Smart Spec Plus Spectrophotometer (BioRad).

### 2.3 Reverse transcription

According to the GoTaq® 2‐Step RT‐qPCR System (Promega) kit instructions, reverse transcription was performed.

### 2.4 Real‐time qPCR

For real‐time qPCR, according to the GoTaq qPCR Master Mix (Promega, Madison, WI, USA) kit instructions, hsa_circ_0001811 was amplified in the Mx3005P real‐time PCR system (Stratagene). circRNAs are different from general linear RNAs, and their primers are specific divergent primers. The primer sequences of hsa_circ_0001811 were 5’‐GGGGCATGTACAATCAGAGATGT‐3’ and 5’‐TTGGGTGGCTTCTGAACTGG‐ 3’, while the primer sequences of the external reference glyceraldehyde 3‐phosphate dehydrogenase (GAPDH) were 5’‐TCGACAGTCAGCCGCATCTTCTTT‐ 3’ and 5’‐ACCAAATCCGTTGACTCCGACCTT‐ 3’. The primers were synthesized by Sangon Biotech (Shanghai, China). The data in this experiment were showed as *Δ*Ct that is inversely proportional to the amount of expression.

### 2.5 Statistical analysis

The data statistical analysis was mainly used GraphPad Prism 6 (GraphPad Software) and SPSS 26.0 (SPSS). Chi‐squared test was used for clinical‐pathological data analysis; paired sample t test was used for analysis hsa_circ_0001811 expression level between gastric cancer tissues and adjacent tissues, and plasma of patients with gastric cancer before and after the operation, while independent sample t test was used for unpaired samples. The experimental data were expressed as mean ± standard deviation. All experiments in this study were repeated three times. When *P* < .05, the difference was considered as statistically significant.

## 3 RESULTS

### 3.1 Determination of specific primers for hsa_circ_0001811

**Figure 1:**
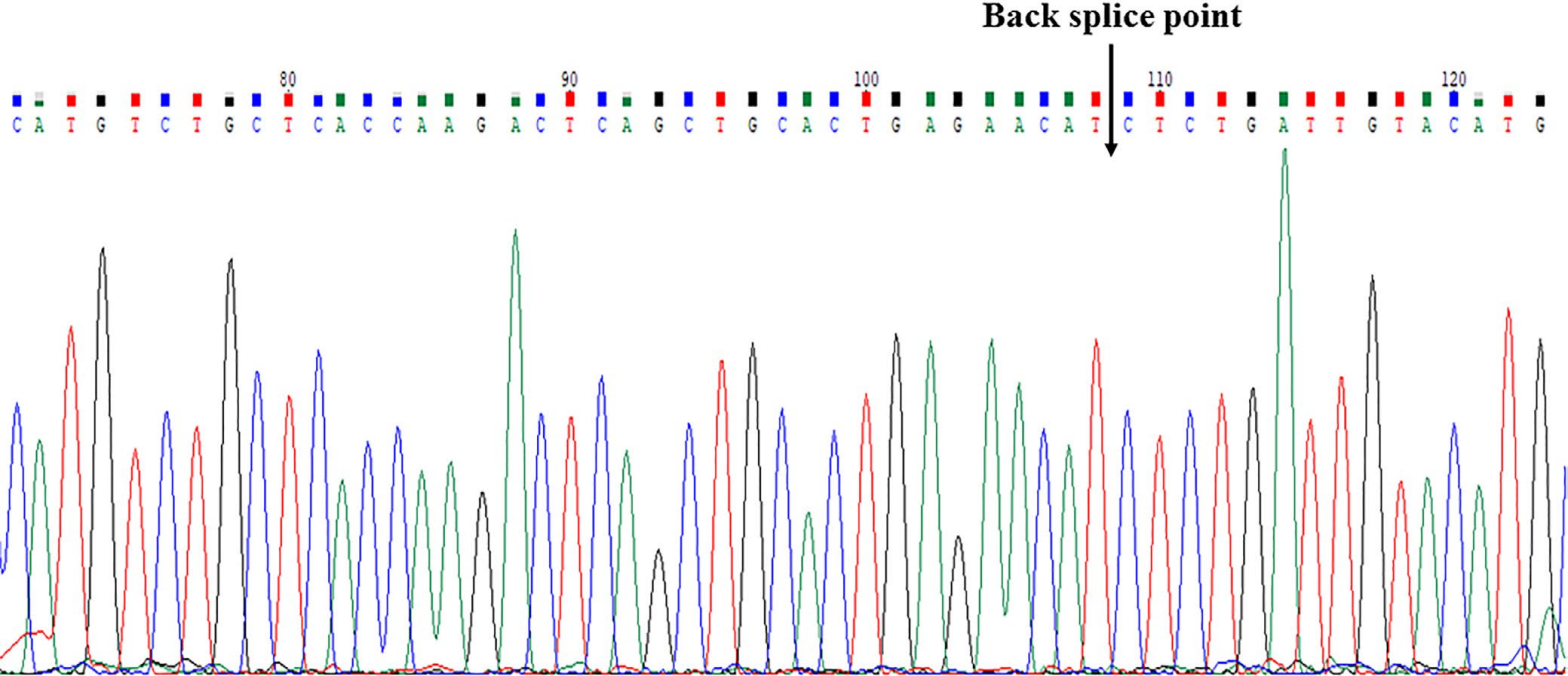
Sanger sequence results of qRT‐PCR products of hsa_circ_0001811

PCR products were sequenced by BGI (Shanghai, China). Sanger sequencing results are shown in Figure 1. After comparison with the circBase, the PCR product sequence is consistent with the target circRNA; that is, this primer is a specific primer of hsa_circ_0001811.

### 3.2 Hsa_circ_0001811 was lowly expressed in gastric cancer tissues and plasma from patients with gastric cancer

**Figure 2:**
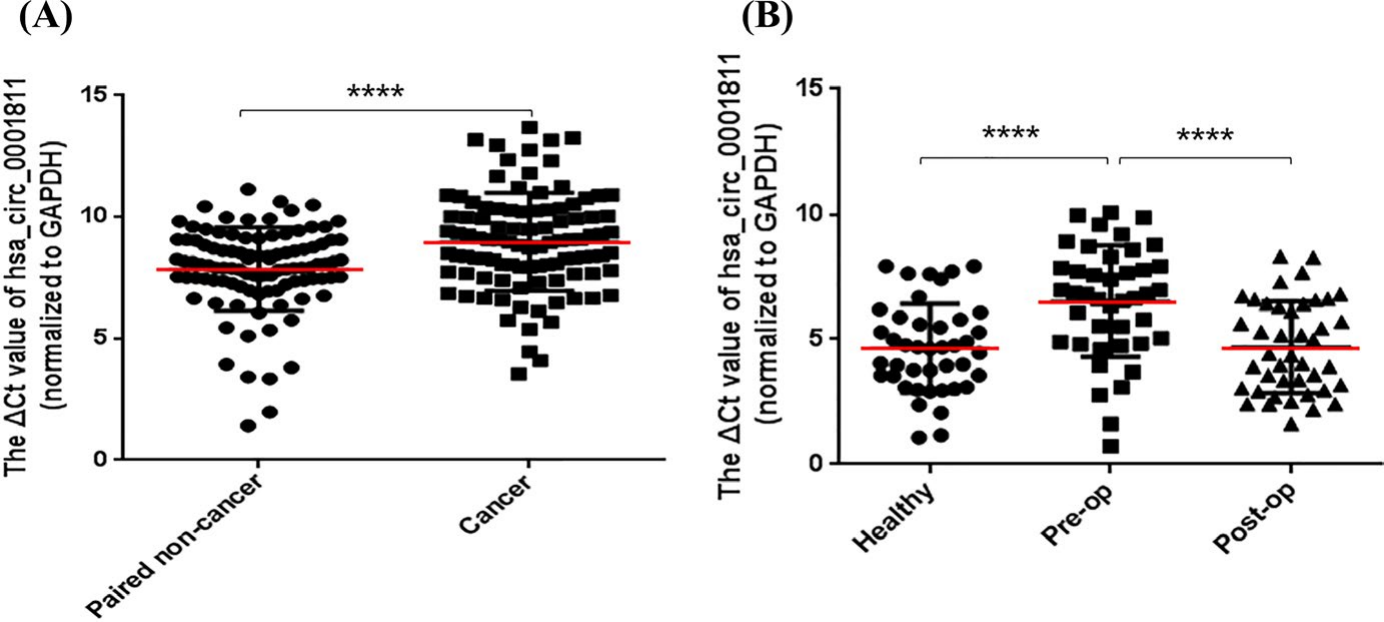
Expression levels of hsa_circ_0001811 in gastric cancer tissues and plasma. The expression levels of hsa_circ_0001811 were significantly downregulated in gastric cancer tissues and plasma. Larger *Δ*C_t_ value indicates lower expression. ^****^ *P* < .0001, A: n = 100, B: n = 42

We used RT‐PCR to detect the expression levels of hsa_circ_0001811 in 100 pairs of gastric cancer tissues and adjacent tissues, and fasting plasma of 42 gastric cancer patients one day before operation and ten days after operation and healthy volunteers. The results showed that compared with paired non‐cancer tissues, hsa_circ_0001811 was low expressed in gastric cancer tissues (Figure 2A); the expression of plasma specimens one day before surgery in gastric cancer patients was lower than those in ten days after surgery and plasma samples of healthy volunteers (Figure 2B).

### 3.3 Potential diagnostic value of hsa_circ_0001811 in gastric cancer

**Figure 3:**
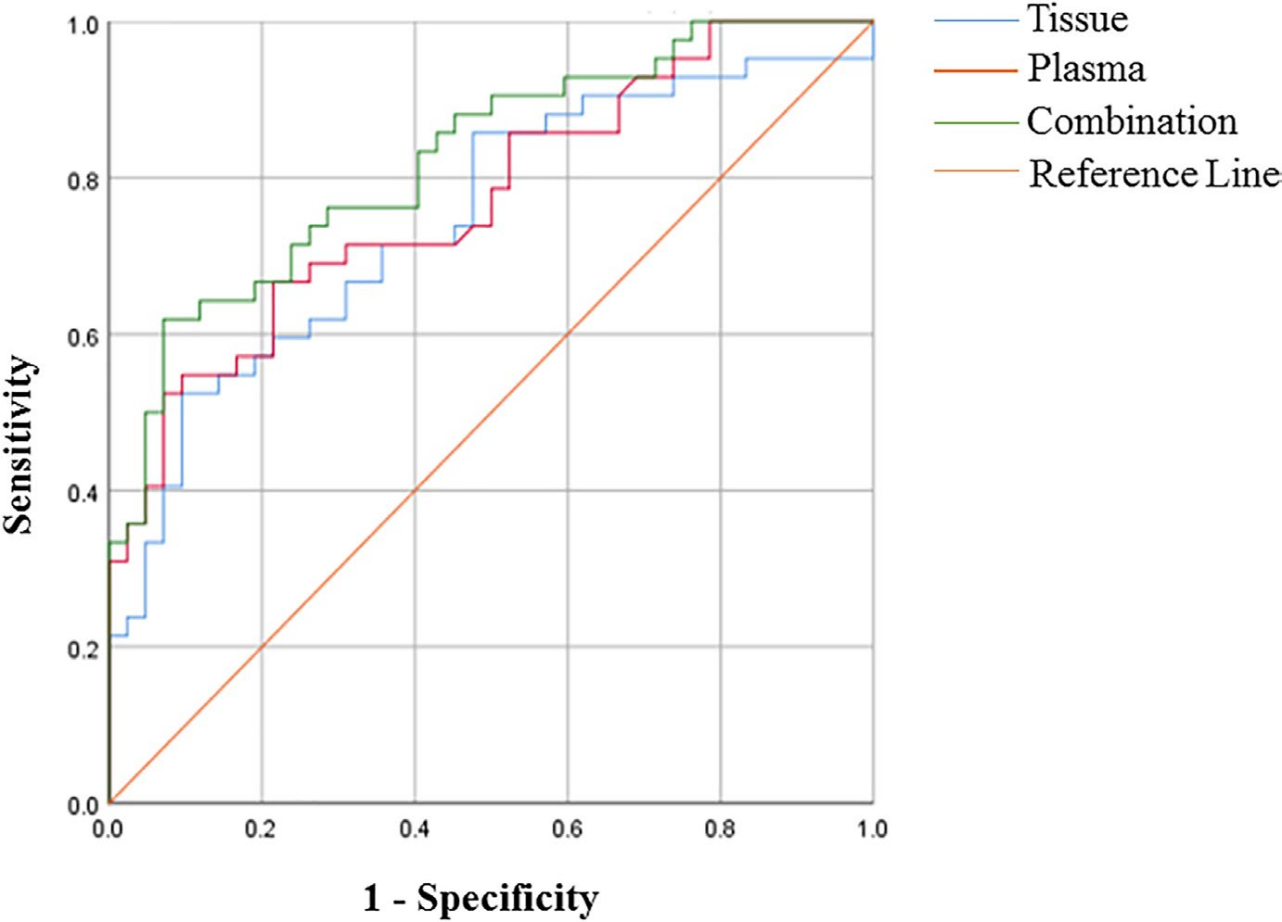
ROC curve of hsa_circ_0001811

The above experimental results prove that the expression of hsa_circ_0001811 in gastric cancer tissues and adjacent tissues, plasma of patients with gastric cancer, and healthy volunteers are significantly different (Figure 2). Therefore, we first draw the ROC curve of hsa_circ_0001811 in tissues and plasma and the combination, and obtained the area under ROC curve (AUC), specificity and sensitivity. As shown in Figure 3, the AUC of hsa_circ_0001811 in gastric cancer tissues was 0.658, while the AUC in plasma was 0.747. When tissues and plasma were combined used, the AUC increased to 0.824, further showing the higher diagnostic value of hsa_circ_0001811.

Then, to further explore its clinical diagnostic value, the relationship between the differential expression of hsa_circ_0001811 in gastric cancer tissues or plasma and the clinical‐pathological factors of gastric cancer patients was analyzed. As shown in Table 1, tissue hsa_circ_0001811 expression level was related to CEA (*P* = .0347), tissue differentiation (*P* = .0138), and lymph node metastasis (*P* = .0234), while plasma hsa_circ_0001811 level (Table 2) was related to CA19‐9 (*P* = .0278), lymph node metastasis (*P* = .0469) and distant metastasis (*P* = .0384), and age (*P* = .0085). These results indicate that the lower the expression level of hsa_circ_0001811 in tissues or plasma, the more obvious these indicators involved.

**Table 1:** The relationships between the expression levels of hsa_circ_0001811 (*Δ*C_t_) in tissues and the clinicopathological factors of patients with gastric cancer

Characteristics	n	High (%)	Low (%)	*P*‐value
All cases	100 (100)	50	50	
Gender				
Male	80 (80.00)	38 (76.00)	40 (80.00)	.6292
Female	20 (20.00)	12 (24.00)	10 (20.00)	
Age (year)				
<60	22 (22.00)	14 (28.00)	8 (16.00)	.1475
≥60	78 (78.00)	36 (72.00)	42 (84.00)	
CEA				
Positive	87 (87.00)	47 (94.00)	40 (80.00)	**.0374**
Negative	13 (13.00)	3 (6.00)	10 (20.00)	
CA19‐9				
Positive	56 (56.00)	24 (48.00)	32 (64.00)	.1070
Negative	44 (44.00)	26 (52.00)	18 (36.00)	
Differentiation				
Well	12 (12.00)	10 (20.00)	2 (4.00)	**.0138**
Moderate‐Poor	88 (88.00)	40 (80.00)	48 (96.00)	
TNM stage				
0 & I	13 (13.00)	13 (26.00)	10 (20.00)	.2729
II	27 (27.00)	16 (32.00)	11 (22.00)	
III & IV	50 (50.00)	21 (42.00)	29 (58.00)	
Invasion				
Tis & T1	22 (22.00)	11 (22.00)	11 (22.00)	.8724
T2 & T3	58 (58.00)	30 (60.00)	28 (56.00)	
T4	20 (20.00)	9 (18.00)	11 (22.00)	
Lymphatic metastasis				
N0	47 (47.00)	20 (40.00)	27 (54.00)	**.0234**
N1 & N2	26 (26.00)	19 (38.00)	7 (14.00)	
N3	27 (27.00)	11 (22.00)	16 (32.00)	
Tumor size (cm)				
<5	28 (28.00)	15 (30.00)	13 (26.00)	.6560
≥5	72 (72.00)	35 (70.00)	37 (74.00)	
Distal metastasis				
M0	83 (83.00)	41 (82.00)	42 (84.00)	.7901
M1	17 (17.00)	9 (18.00)	8 (16.00)	

The bold values indicate statistically significant.

**Table 2:** The relationships between plasma hsa_circ_0001811 level (*Δ*C_t_) and the clinicopathological factors of patients with gastric cancer

Characteristics	n	High (%)	Low (%)	*P*‐value
All cases	42 (100)	21	21	
Gender				
Male	33 (78.57)	16 (71.43)	17 (80.95)	.7069
Female	9 (21.43)	5 (23.81)	4 (19.05)	
Age (year)				
<60	9 (21.43)	1 (4.76)	8 (38.10)	**.0085**
≥60	33 (78.57)	20 (95.24)	13 (61.90)	
CEA				
Positive	38 (90.48)	18 (85.71)	20 (95.24)	.2931
Negative	4 (9.52)	3 (14.29)	1 (4.76)	
CA19‐9				
Positive	25 (59.52)	9 (42.86)	16 (76.19)	**.0278**
Negative	17 (40.48)	12 (57.14)	5 (23.81)	
Differentiation				
Well	4 (9.52)	1 (4.76)	3 (14.29)	.2931
Moderate‐Poor	38 (90.48)	20 (95.24)	18 (85.71)	
TNM stage				
0 & I	9 (21.43)	5 (23.81)	4 (19.05)	.7819
II	12 (28.57)	5 (23.81)	7 (33.33)	
III & IV	21 (50.00)	11 (52.38)	10 (47.62)	
Invasion				
Tis & T1	7 (16.67)	4 (19.05)	3 (14.29)	.6557
T2 & T3	19 (45.24)	15 (71.43)	14 (66.66)	
T4	6 (38.09)	2 (9.52)	4 (19.05)	
Lymphatic metastasis				
N0	13 (30.95)	4 (19.05)	9 (42.86)	**.0469**
N1 & N2	22 (52.38)	15 (71.43)	7 (33.33)	
N3	7 (16.67)	2 (9.52)	5 (23.81)	
Tumor size(cm)				
<5	11 (26.19)	5 (23.81)	6 (28.57)	.7256
≥5	31 (73.81)	16 (76.19)	15 (71.43)	
Distal metastasis				
M0	35 (83.33)	15 (71.43)	20 (95.24)	**.0384**
M1	7 (16.67)	6 (28.57)	1 (4.76)	

The bold values indicate statistically significant.

## 4 DISCUSSION

CircRNAs were first identified as faulty spliced transcripts in viruses.^12^, ^13^ With the development of RNA detection technology, more and more circRNAs have appeared in the public, attracting widespread attention.^14^ At the same time, a growing number of studies have reported that circRNAs are a new type of RNAs that can be stable in tissues and plasma.^15^, ^16^, ^17^ In particular, it should be noted that circRNAs can participate in the development of many diseases and regulate the course of diseases, especially tumors.^18^, ^19^, ^20^, ^21^ Therefore, circRNAs as a new diagnostic marker have become the top priority in the research.^22^, ^23^

CircRNAs can be divided into three types, exon type ecircRNAs,^24^ intron type ciRNAs,^25^ exon‐intron type also known as mixed type EIciRNAs,^26^ the most of which is ecircRNAs. Different types of circRNAs are distributed in different positions,^27^ ecircRNAs are mostly distributed in the cytoplasm, while ciRNAs and EIciRNAs are mostly distributed in the nucleus.^28^, ^29^ Different distributions of circRNAs can play different roles. At present, the functions of circRNAs are not yet known. Possible functions include acting as a miRNAs sponge,^30^ interacting with RNA binding proteins,^31^ encoding proteins,^32^, ^33^ and regulating transcription.^34^ One of the most classic is acting as a miRNAs sponge. The earliest discovery was the cerebellar degeneration‐related protein 1 antisense transcript (CDR1as), a representative circRNA containing approximately 70 miR‐7 binding sites, which classically interpreted the sponge effect of the final competitive inhibition, thereby affecting the target gene expression.^35^ Due to the special structure of circRNAs, compared with linear RNAs, it can be stable in tissues and plasma, and has tissue and timing specificity of expression, which also provides a basis and clear direction for our research.

In this study, we focused on hsa_circ_0001811, which belongs to ecircRNAs and is composed of two exons. We first designed its specific primers for hsa_circ_0001811. On this basis, we expanded the tissue sample size to 100 pairs. The results indicated that hsa_circ_0001811 was lowly expressed in gastric cancer tissues compared to adjacent non‐cancer tissues (Figure 2A). As a result of it is time‐consuming and labor‐intensive to take cancer tissues for clinical diagnosis, plasma is relatively convenient. Therefore, 42 preoperative and postoperative plasma samples of gastric cancer patients and 42 healthy volunteer plasma samples were further detected. The results demonstrated that compared with 10 days after surgery and healthy volunteers, the level of plasma hsa_circ_0001811 was lowered in patients with gastric cancer one day before surgery (Figure 2B). To further explore its diagnostic value, we made a ROC curve. The combination of the AUC increased to 0.824 (Figure 3). The sensitivity and specificity in gastric tissues were 0.50 and 0.81, respectively, while plasma was 0.91 and 0.52, respectively. The diagnostic value is higher than the existing clinical CEA and CA19‐9 for gastric cancer diagnosis. Then, we analyzed the relationship between the levels of hsa_circ_0001811 in tissues or plasma and clinical‐pathological data of gastric cancer patients. We found that the expression levels of hsa_circ_0001811 in gastric cancer tissues were related to CEA, tissue differentiation, and lymph node metastasis (Table 1), while plasma hsa_circ_0001811 level was related to CA19‐9, lymph node metastasis, and distant metastasis and age (Table 2). These results indicate that it may become a new diagnostic biomarker for gastric cancer.

Because circRNAs are more stable and highly conservative in tissues and plasma than other RNAs, such as long non‐coding RNA (lncRNA) and miRNA, these characteristics make circRNAs an effective new biomarker for non‐invasive diagnosis of gastric cancer.

## 5 CONCLUSION

In conclusion, in this study, the data we obtained showed that hsa_circ_0001811 is low expressed in gastric cancer tissues and plasma, and its expression level can be detected by qRT‐PCR. These findings indicate that hsa_circ_0001811 may be a new potential biomarker for the diagnosis of gastric cancer.
